# Contribution to the ongoing discussion on fluoride toxicity

**DOI:** 10.1007/s00204-021-03072-6

**Published:** 2021-06-06

**Authors:** Sabine Guth, Stephanie Hüser, Angelika Roth, Gisela Degen, Patrick Diel, Karolina Edlund, Gerhard Eisenbrand, Karl-Heinz Engel, Bernd Epe, Tilman Grune, Volker Heinz, Thomas Henle, Hans-Ulrich Humpf, Henry Jäger, Hans-Georg Joost, Sabine E. Kulling, Alfonso Lampen, Angela Mally, Rosemarie Marchan, Doris Marko, Eva Mühle, Michael A. Nitsche, Elke Röhrdanz, Richard Stadler, Christoph van Thriel, Stefan Vieths, Rudi F. Vogel, Edmund Wascher, Carsten Watzl, Ute Nöthlings, Jan G. Hengstler

**Affiliations:** 1 Department of Toxicology, Leibniz Research Centre for Working Environment and Human Factors (IfADo), Dortmund, Germany; 2 Department of Molecular and Cellular Sports Medicine, Institute of Cardiovascular Research and Sports Medicine, German Sport University Cologne, Cologne, Germany; 3 Kühler Grund 48/1, 69126 Heidelberg, Germany; 4 Department of General Food Technology, School of Life Sciences, TU Munich, Freising, Germany; 5 Institute of Pharmacy and Biochemistry, University of Mainz, Mainz, Germany; 6 Department of Molecular Toxicology, German Institute of Human Nutrition (DIfE), Nuthetal, Germany; 7 German Institute of Food Technologies (DIL), Quakenbrück, Germany; 8 Department of Food Chemistry, TU Dresden, Dresden, Germany; 9 Institute of Food Chemistry, Westfälische Wilhelms-Universität Münster, Münster, Germany; 10 Institute of Food Technology, University of Natural Resources and Life Sciences (BOKU), Vienna, Austria; 11 Department of Experimental Diabetology, German Institute of Human Nutrition (DIfE), Nuthetal, Germany; 12 Department of Safety and Quality of Fruit and Vegetables, Max Rubner-Institut, Federal Research Institute of Nutrition and Food, Karlsruhe, Germany; 13 Department of Food Safety, Bundesinstitut für Risikobewertung (BfR), Berlin, Germany; 14 Department of Toxicology, University of Würzburg, Würzburg, Germany; 15 Department of Food Chemistry and Toxicology, Faculty of Chemistry, University of Vienna, Vienna, Austria; 16 Department of Psychology and Neurosciences, Leibniz Research Centre for Working Environment and Human Factors (IfADo), Dortmund, Germany; 17 Department of Neurology, University Medical Hospital Bergmannsheil, Ruhr-University, Bochum, Germany; 18 Department of Experimental Pharmacology and Toxicology, Federal Institute for Drugs and Medical Devices (BfArM), Bonn, Germany; 19 Institute of Food Safety and Analytic Sciences, Nestlé Research Centre, Lausanne, Switzerland; 20 Paul-Ehrlich-Institut, Langen, Germany; 21 Lehrstuhl für Technische Mikrobiologie, TU Munich, Freising, Germany; 22 Department of Ergonomics, Leibniz Research Centre for Working Environment and Human Factors (IfADo), Dortmund, Germany; 23 Department of Immunology, Leibniz Research Centre for Working Environment and Human Factors (IfADo), Dortmund, Germany; 24 Department of Nutrition and Food Sciences, Nutritional Epidemiology, Rheinische Friedrich-Wilhelms University Bonn, Bonn, Germany

## Abstract

Since the addition of fluoride to drinking water in the 1940s, there have been frequent and sometimes heated discussions regarding its benefits and risks. In a recently published review, we addressed the question if current exposure levels in Europe represent a risk to human health. This review was discussed in an editorial asking why we did not calculate benchmark doses (BMD) of fluoride neurotoxicity for humans. Here, we address the question, why it is problematic to calculate BMDs based on the currently available data. Briefly, the conclusions of the available studies are not homogeneous, reporting negative as well as positive results; moreover, the positive studies lack control of confounding factors such as the influence of well-known neurotoxicants. We also discuss the limitations of several further epidemiological studies that did not meet the inclusion criteria of our review. Finally, it is important to not only focus on epidemiological studies. Rather, risk analysis should consider all available data, including epidemiological, animal, as well as in vitro studies. Despite remaining uncertainties, the totality of evidence does not support the notion that fluoride should be considered a human developmental neurotoxicant at current exposure levels in European countries.

## Introduction

Since the 1940s, fluoride has been added to drinking water in many countries as a means of caries prophylaxis. Fluoride prevents caries at low exposure levels, whereas, excessive fluoride exposure causes dental and skeletal fluorosis in humans, and developmental toxicity in animals. Based on this background, the European Food Safety Authority (EFSA) defined an adequate intake (AI) level for fluoride of 50 µg/kg b.w. at which the caries preventive effect approached its maximum whilst the risk of dental fluorosis approached its minimum (EFSA 2013). In recent years, the benefits and risks of fluoride exposure to the general population, e.g. by drinking water, fluoridated salt or dental care products, have been heavily debated, and special focus is set on potential adverse health effects, such as neurodevelopmental toxicity.

## What type of data is needed to assess fluoride developmental neurotoxicity?

To adequately address potential human health concerns caused by exposure to fluoride, the available evidence from all sources should be included. Thus, it is crucial to critically review the evidence from epidemiological, as well as from animal and in vitro studies. Recently, we published a comprehensive review considering the available data from all the study types mentioned above, particularly focusing on developmental toxicity (Guth et al. 2020). Another factor to consider when assessing the potential health risks of fluoride is the expected level of exposure. The focus of our review was on studies investigating the developmental effects of fluoride levels in drinking water in the range of community water fluoridation (CWF) of 0.7–1.0 mg/L, as well as naturally occurring exposure scenarios in Europe which generally do not exceed the AI defined by EFSA. Since our aim was to evaluate whether fluoride exposure in European countries is of potential health concern, we did not address other exposure scenarios, e.g. in areas with endemically occurring high fluoride concentrations in ground and drinking water.

In comparison, other reviews evaluating a potential developmental toxicity of fluoride (e.g. Choi et al. 2012; Grandjean 2019; Grandjean and Landrigan 2014) (i) focused on the evidence from epidemiological studies, but did not include experimental evidence, and/or (ii) included results from endemically high fluoride areas. Thus, it is important to recognize that our review, in comparison to others recently published on fluoride toxicity, aimed to address different questions, and this is reflected by the application of different inclusion criteria used. It is therefore not surprising that conclusions drawn by the authors differ in some respects.

Below, evidence from animal, in vitro and epidemiological studies is briefly summarized primarily focusing on European exposure scenarios as discussed in our review by Guth et al. (2020).

## Evidence from animal studies

Chronic toxicity studies in rats, mice, and rabbits that focused on systemic effects of fluoride resulted in Lowest-Observed-Adverse-Effect Levels (LOAELs) ranging between 4.3 and 7.6 mg/kg b.w./day fluoride, and no-observed-adverse-effect levels (NOAELs) between 2.5 and 7.6 mg/kg b.w./day fluoride. Four well-conducted developmental toxicity studies (Collins et al. 2001, 1995; Heindel et al. 1996) are available which are in accordance with standard guidelines, used adequate numbers of animals, and administered sodium fluoride in drinking water. These studies resulted in NOAELs of 8.5–13.7 mg/kg b.w./day fluoride for rats and rabbits. It should be noted that the influence of specific fluoride doses on plasma levels may vary between different species. For example, it has been suggested that approximately fivefold higher doses in drinking water might be required for rats to achieve serum concentrations similar to those in humans (Dunipace et al. 1995; NRC 2006). However, it must also be taken into account that numerous variables could influence these relationships in both animal and human studies and the factor to calculate plasma concentrations is largely uncertain, in part because it could change with age or duration of exposure (NRC 2006).

To our knowledge, there are currently no further developmental studies that were performed according to standard guidelines. A search of the literature published between 2005 and 2018 revealed a number of animal studies that reported an effect of fluoride exposure on various endpoints in offspring during development (see (Guth et al. 2020)). We reviewed the quality of these studies and identified various limitations (see Box 1) that hamper their interpretation, thus reducing their value for risk assessment.

**Figure:**
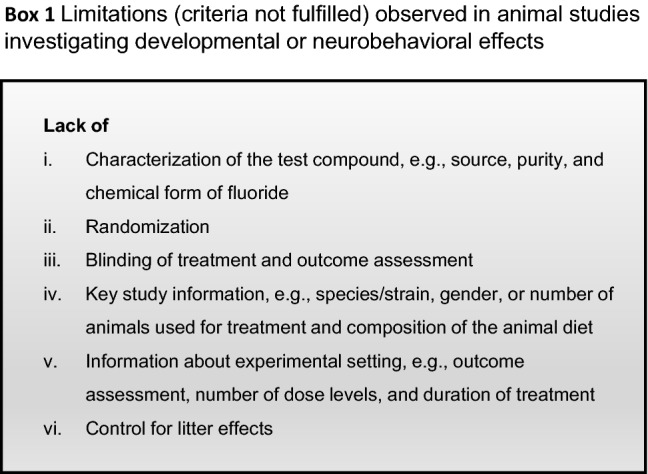

Studies investigating neurobehavioral toxicity in animals produced conflicting results (NTP 2016). A systematic review by the US National Toxicology Program (NTP) reported a low to moderate level of evidence of adverse effects on learning and memory in rats and mice exposed to fluoride concentrations substantially higher than 0.7 mg/L (NTP 2016). After the publication of the NTP report in 2016, several studies became available that investigated the impact of fluoride exposure on memory and learning in experimental animals. We reviewed the quality of the available studies and found that only two fulfilled the criteria listed in Box 1, but nevertheless still had limitations (McPherson et al. 2018; Pulungan et al. 2018). In both studies, no exposure-related differences in motor, sensory, or learning and memory performance were observed with the exposure levels investigated (up to 20 mg fluoride/L or 9 mg fluoride/kg b.w./day, respectively). Of note, one study (McPherson et al. 2018) primarily investigated the influence of fluoride exposure during development on neurobehavioral aspects. The other 11 studies identified in our search had various and strong limitations, and did not meet key quality criteria discussed in detail in our review (see (Guth et al. 2020)).

## Human exposure in relation to adverse effects in animal experiments

The mean intake of fluoride from water, food, beverages and oral hygiene products in European populations is usually below the AI recommended by EFSA. Recently, there has been some debate as to whether exposure in the range of the AI, i.e. 50 µg fluoride/kg b.w. /day, is sufficient to cause an increased risk of adverse effects in humans. It has also been suggested that fluoride, at current exposure levels, should be categorized as a human developmental neurotoxicant, and be placed in the same category as lead, methyl mercury, arsenic and polychlorinated biphenyls (Grandjean 2019; Grandjean and Landrigan 2014). To evaluate the situation, we calculated a margin of exposure (MoE) between doses showing no adverse effects in animal studies and the AI (Guth et al. 2020). The lowest NOAEL for systemic toxicity from a well-designed chronic animal study was 2.5 mg/kg b.w./day. The lowest NOAEL for developmental toxicity was 8.5 mg/kg b.w./day. Compared to the AI of 50 µg/kg/day, the margin of exposure (MoE) is ~ 50 (systemic toxicity) or ~ 170 (developmental toxicity), which are high MoEs.

## Evidence from in vitro studies

Recent findings suggest that in vitro data should also be considered in the risk evaluation of chemicals (Godoy et al. 2013; Leist 2017). Therefore, we compared the highest reported fluoride concentrations in plasma of healthy individuals (3 µM; summarized by Guth et al. 2020; e.g. Rugg-Gunn et al. 2011) to cell culture medium concentrations causing cytotoxic effects in neuronal and stem cells of rodent and human origin, which occurred at ~ 1 mM in most studies (range: 0.1–4 mM) (Guth et al. 2020). This results in a ratio of ~ 300, which demonstrates that human plasma concentrations of fluoride are far below cytotoxic levels.

## Evidence from epidemiological studies

Since our review (Guth et al. 2020) addressed the exposure scenarios relevant for European countries, we focused on epidemiological studies conducted in non-endemic fluoride areas or areas with CWF. Furthermore, we based our assessment on prospective studies in which cohorts were followed over a period of time (see inclusion criteria, Box 3). Two prospective cohort studies conducted in CWF areas that considered possible confounding factors (Broadbent et al. 2015; Green et al. 2019, Box 5) were included in our evaluation, and both reported conflicting results. In our review, we also noted that the majority of epidemiological studies conducted in areas with endemically occurring high fluoride levels in ground and drinking water reported an association between lower measures of intelligence and high fluoride exposure.

Other reviews (e.g. Choi et al. 2012; Grandjean 2019; Grandjean and Landrigan 2014) did not only focus on community water fluoridation and prospective cohort studies, but also included cross-sectional epidemiological studies, as well as studies performed in areas with endemically occurring high fluoride concentrations in drinking water. In these reviews, it was concluded that recent epidemiological evidence suggests that elevated fluoride intake during early development can result in considerable IQ deficits (Grandjean 2019).

While the present letter was under review, an article on a retrospective cohort study was published performed by a research institute under the Swedish Ministry of Employment (Institute for Evaluation of Labour Market and Education Policy; IFAU), which estimated a zero effect on cognitive ability for fluoride levels in Swedish drinking water (Aggeborn and Oehman 2021). This article is based on data of a comprehensive retrospective cohort study already discussed in our previous review (Aggeborn and Oehman 2017; see also Guth et al. 2020).

## Limitations of epidemiological studies and inclusion criteria

Our analysis of the epidemiological studies repeatedly identified the limitations summarized in Box 2. In line with our goal to assess possible effects of fluoride at current exposure levels in Europe, we used the inclusion criteria summarized in Box 3. However, in a recent editorial (Spittle 2020), the author wrote that we omitted specific studies in the epidemiological section of our manuscript (Guth et al. 2020). This was indeed the case, because these studies did not meet our inclusion criteria. We provide a standardized profile and brief discussion of these studies with their strength and limitations, while simultaneously addressing the comments of Spittle (2020) (Box 4). These studies do not change the overall conclusion that the totality of currently available scientific evidence does not support the concept that fluoride should be assessed as a human developmental neurotoxicant at the current exposure levels in Europe. Furthermore, the authors of the studies (Box 4) were aware of these limitations and usually addressed them in the corresponding discussions.

**Figure:**
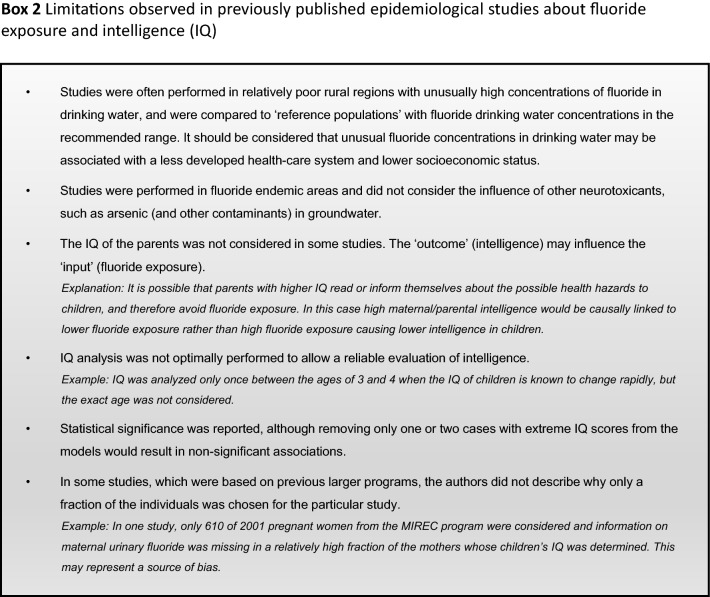

**Figure:**
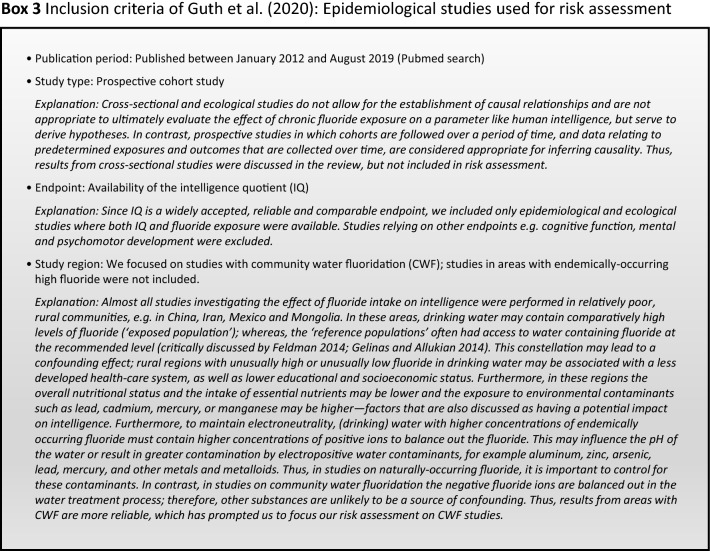

**Figure:**
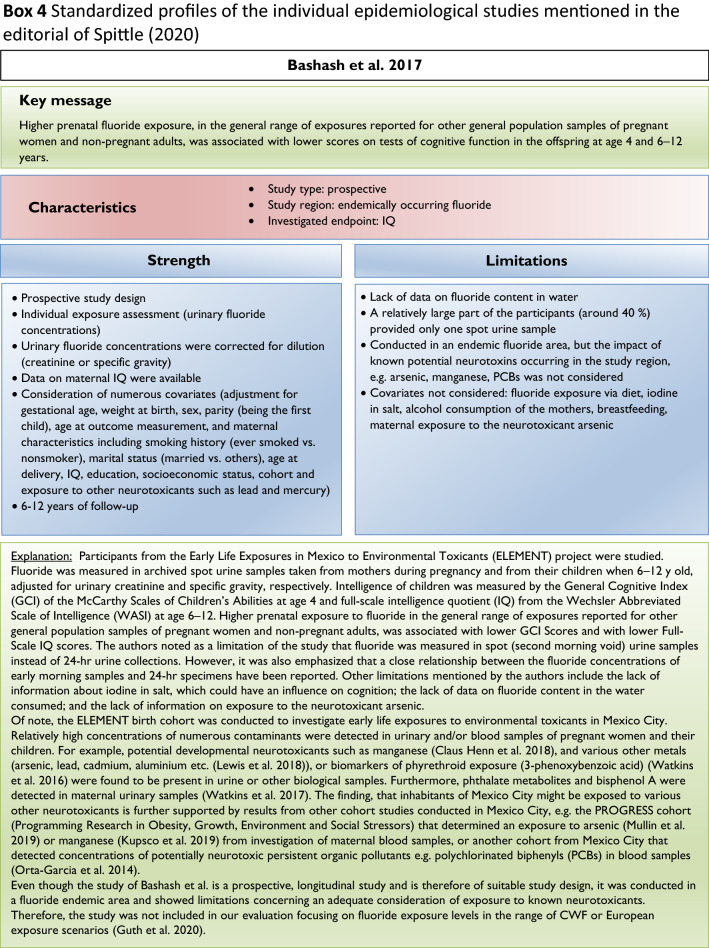


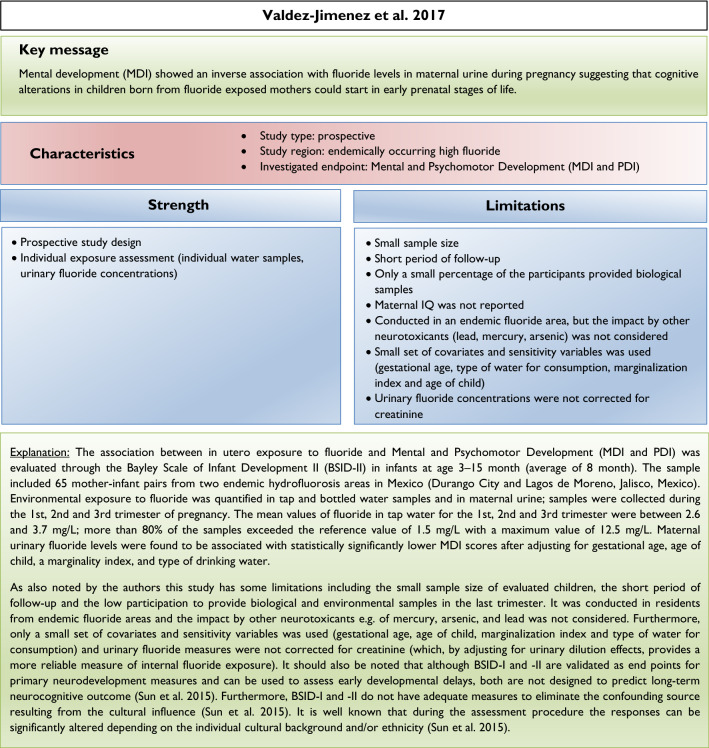

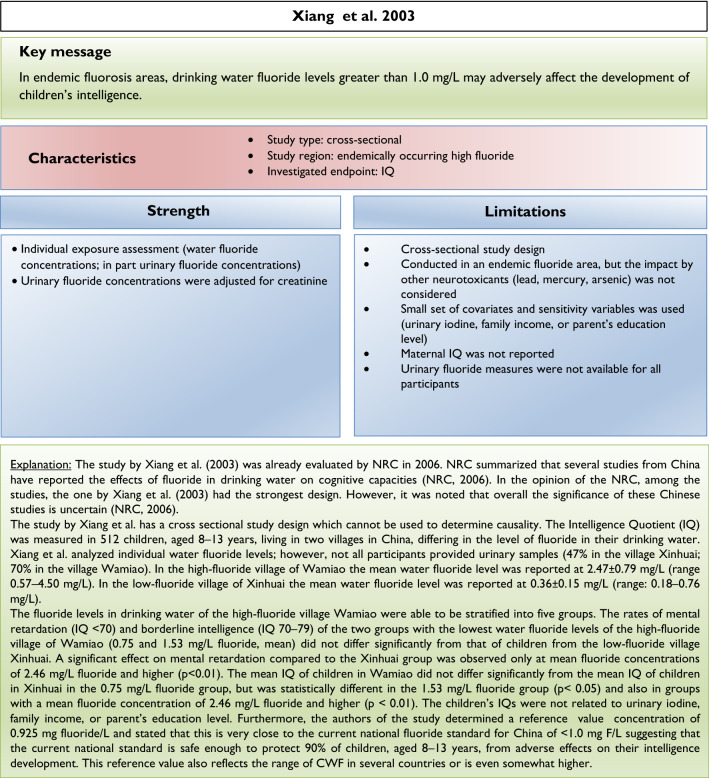

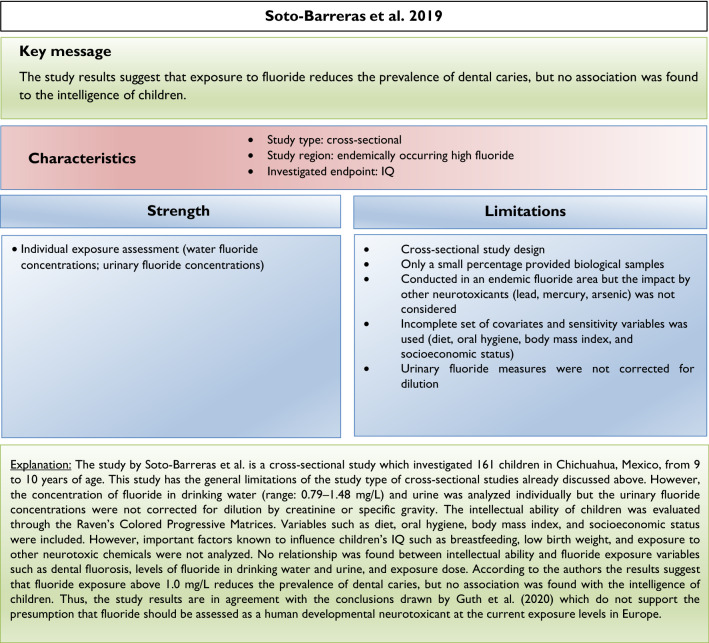

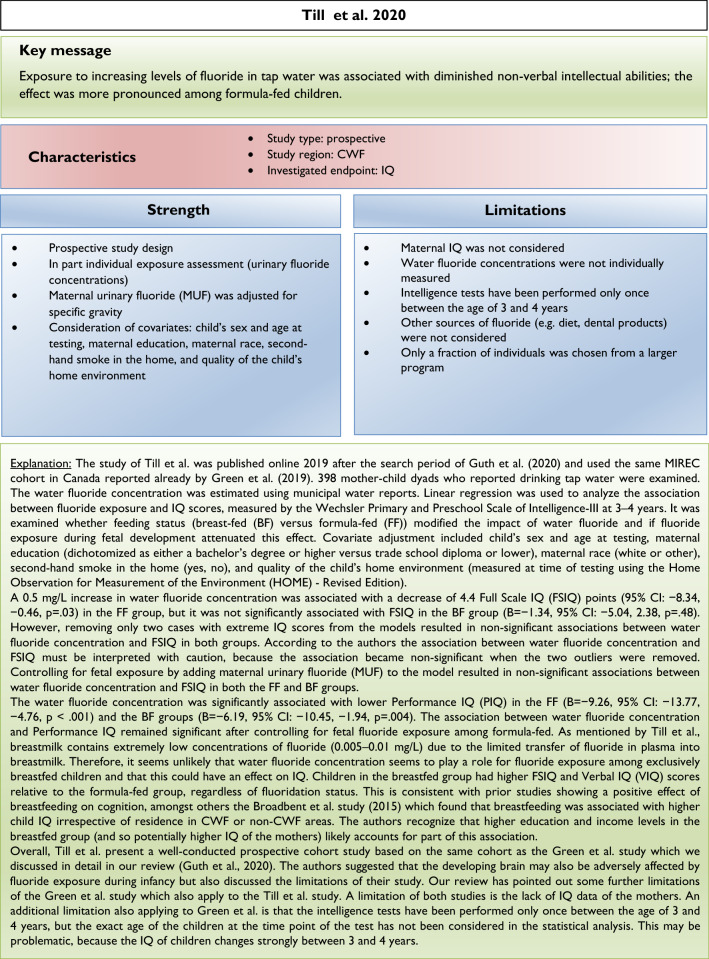


## Discrepancy between experimental and epidemiological evidence

We observed a discrepancy between experimental and epidemiological evidence, which may be explained by deficiencies that were inherent to most of the current epidemiological studies, e.g. insufficient consideration of potential confounders. The majority of epidemiological studies which reported an association between lower measures of intelligence and high fluoride exposure was conducted in areas with endemically occurring high fluoride levels in ground and drinking water. In contrast, the experimental evidence suggests that current exposure to fluoride, even for individuals with relatively high fluoride intake, is clearly below levels that have led to adverse effects in vitro or in animals.

## Reasons why it is problematic to calculate bench mark doses for humans (BMD)

A main criticism of our review was that we made ‘*no attempt to calculate the threshold for fluoride neurotoxicity using the standard benchmark dose method.’…’Grandjean singled out three prospective studies, two in 2017 from Mexico by Valdez Jiménez *et al*. and Bashash *et al*. and one in 2018 from Canada by Green *et al*., … to estimate the tentative benchmark dose (BMD) values.*’ (Spittle 2020).

Grandjean used the regression coefficients and their standard deviations as provided in the published reports to estimate tentative BMD values. A BMDL of about 0.2 mg/L or below was suggested (Grandjean 2019), which was similar to the result calculated from a previous study (Xiang et al. 2003) by Hirzy et al. (Hirzy et al. 2016) (for a brief discussion of studies calculating BMDs for humans see Box 6). It should be considered that even without fluoridation, the fluoride concentration in drinking water in Europe often ranges around 0.5 mg/L and is therefore higher than the BMDL of 0.2 mg/L derived by Grandjean et al. (2019). It was concluded that the benchmark dose of fluoride neurotoxicity is clearly below commonly occurring fluoride exposure levels.

**Fig. 1:**
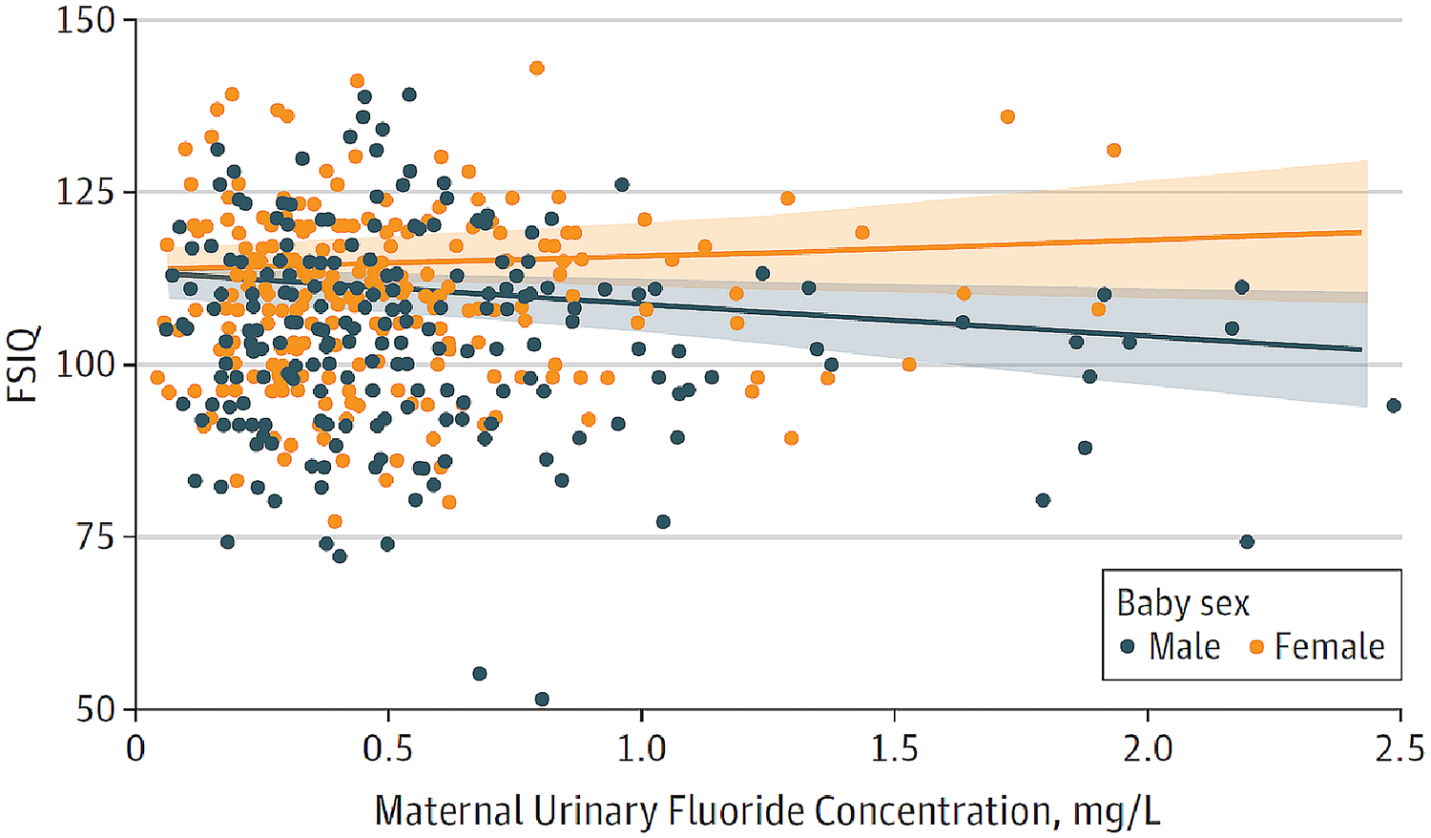
Correlation of maternal urinary fluoride concentration and full-scale IQ (FSIQ), reproduced from Green et al., 2019. Using this set of data, the authors concluded: “An increase from the 10th to 90th percentile of maternal urinary fluoride was associated with a 3.14 IQ decrement among boys.” (Green et al. 2019). However, because of the relatively high variability of the IQ data, recently calculated benchmark doses of human neurotoxicity (Grandjean 2019) should be treated with caution

We did not follow this approach to calculate the BMD, because the results of such calculations would be questionable due to the inadequate quality of the available input data. It remains unclear why two studies (Bashash et al. 2017; Green et al. 2019) were finally selected to calculate the BMD (Grandjean 2019); whereas, others with a negative result (Broadbent et al. 2015) were omitted. The studies by Valdez Jimenez et al. (2017) and Bashash et al. (2017) have limitations, such as the lack of control of the influence of other neurotoxicants and small sample size (Box 4). Green et al. (2019) openly discussed the limitations of their own study directly in their publication, which are briefly summarized in Box 5. The difficulty to ‘calculate the threshold of fluoride neurotoxicity’ (Spittle 2020) is illustrated using a scatter plot of IQ (FSIQ) versus maternal urinary fluoride, where each dot represents the IQ of a child (Fig. 1; reproduced Fig. 3A from (Green et al. 2019)). The trend line of IQ of the girls slightly increased with higher maternal urinary fluoride, although this effect was not statistically significant. In contrast, a significantly lower IQ was observed for boys, which depended on the two individuals with the highest urinary fluoride (Fig. 1). This difference led to the suggestion that there may be a sex difference in response to fluoride (Grandjean 2019). However, considering the high overall variability of IQ among the children in the study, this interpretation should be done with caution. Rather, further studies are required before such conclusions can be drawn.

**Figure:**
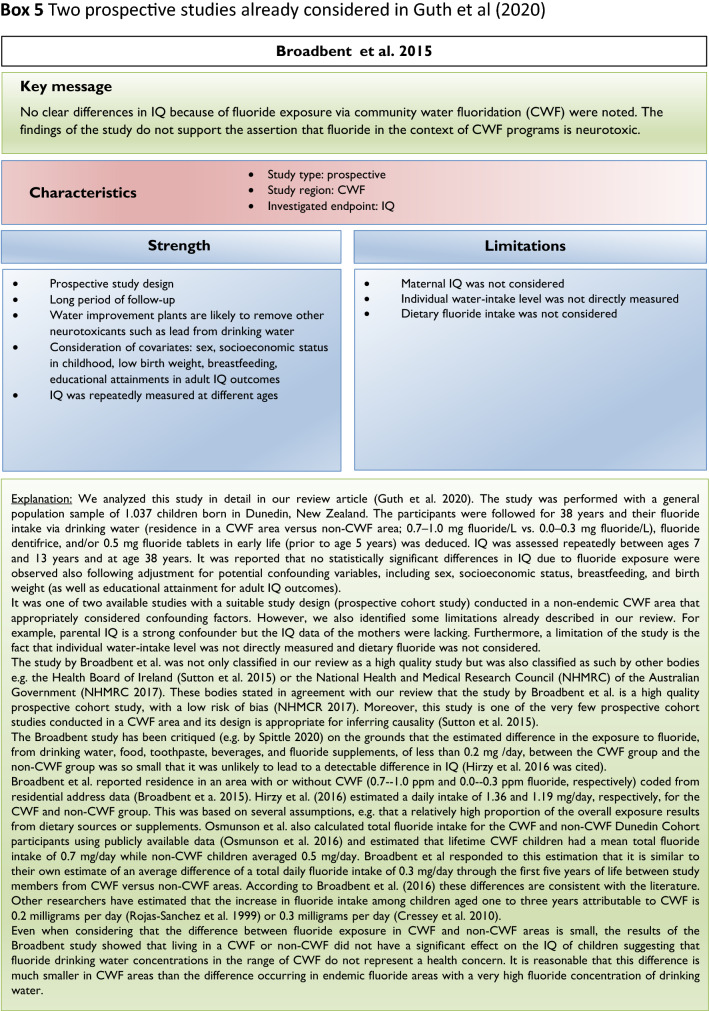


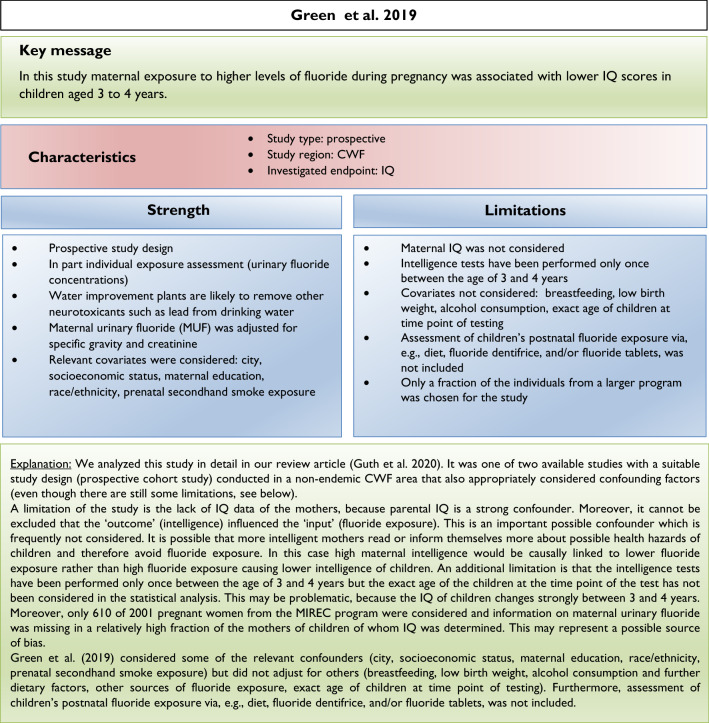

**Figure:**
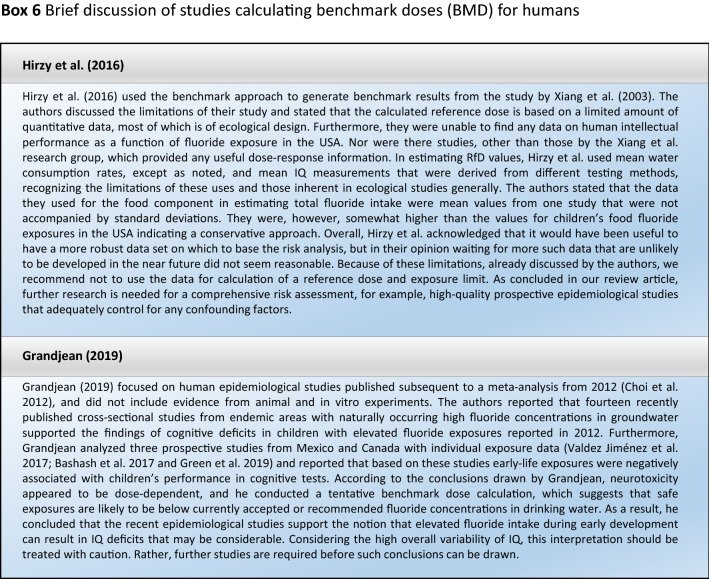

## Conclusion

There are varying opinions on the health effects of high fluoride exposure. Our recent assessment was based on evidence from animal, in vitro and epidemiological studies focusing on exposure scenarios relevant for the population in Europe. Others included epidemiological evidence from endemic areas into their assessment. Moreover, we critically discussed the insufficient consideration of confounding factors and deficiencies of study design and statistical evaluation in available epidemiological studies. Thus, the differences in considered study populations and different standards in evaluating the quality of epidemiological studies may at least in part explain the different assessments. Also, considering the additional studies which did not meet the inclusion criteria of our first review article (see Box 4), we still arrive at the same conclusions: the available epidemiological evidence does not provide sufficient arguments to raise concerns with regard to CWF in the range of 0.7–1.0 mg/L, nor does it justify that fluoride should be categorized as a human developmental neurotoxicant, signifying that it is similarly problematic as lead or methylmercury at current exposure levels. Of course, the conclusions may have to be reconsidered if new comprehensive findings from epidemiological or animal studies are presented.

## Final recommendations

Calculation of a threshold for human fluoride neurotoxicity based on selected epidemiological studies may be problematic since the available data are not considered to be sufficient to perform a dose response assessment. For risk evaluation, it is important to consider all available data, including animal experiments and in vitro studies. Further animal studies and prospective epidemiological studies would be helpful, but should avoid the limitations of previous investigations as summarized in Box 1, Box 2 and described by Guth et al. (2020).

## References

[CR1] Aggeborn L, Oehman M (2017) The effects of fluoride in drinking water. Uppsala: Institute for Evaluation of Labour Market and Education Policy, p. 1–83. https://www.ifau.se/globalassets/pdf/se/2017/wp2017-20-the-effects-of-fluoride-in-the-drinking-water.pdf

[CR2] Aggeborn L, Oehman M (2021) The effects of fluoride in drinking water. Journal of Political Economy 129(2):465–491. 10.1086/711915

[CR3] Bashash M, Thomas D, Hu H (2017). Prenatal fluoride exposure and cognitive outcomes in children at 4 and 6–12 years of age in Mexico. Environ Health Perspect.

[CR4] Broadbent JM, Thomson WM, Ramrakha S (2015). Community water fluoridation and intelligence: prospective study in New Zealand. Am J Public Health.

[CR5] Broadbent JM, Thomson WM, Moffitt TE, Poulton RBroadbent, (2016). Respond. Am J Public Health.

[CR6] Choi AL, Sun G, Zhang Y, Grandjean P (2012). Developmental fluoride neurotoxicity: a systematic review and meta-analysis. Environ Health Perspect.

[CR7] Claus Henn B, Austin C, Coull BA (2018). Uncovering neurodevelopmental windows of susceptibility to manganese exposure using dentine microspatial analyses. Environ Res.

[CR8] Collins TF, Sprando RL, Shackelford ME (1995). Developmental toxicity of sodium fluoride in rats. Food Chem Toxicol.

[CR9] Collins TF, Sprando RL, Black TN (2001). Developmental toxicity of sodium fluoride measured during multiple generations. Food Chem Toxicol.

[CR10] Cressey P, Gaw S, Love J (2010). Estimated dietary fluoride intake for New Zealanders. J Public Health Dent.

[CR11] Dunipace AJ, Brizendine EJ, Zhang W (1995). Effect of aging on animal response to chronic fluoride exposure. J Dent Res.

[CR12] EFSA (2013). Panel on dietetic products, nutrition; scientific opinion on dietary reference values for fluoride. EFSA J.

[CR13] Feldman V (2014). Neurodevelopmental toxicity: still more questions than answers. Lancet Neurol.

[CR14] Gelinas J, Allukian M (2014). Neurodevelopmental toxicity: still more questions than answers. Lancet Neurol.

[CR15] Godoy P, Hewitt NJ, Albrecht U (2013). Recent advances in 2D and 3D in vitro systems using primary hepatocytes, alternative hepatocyte sources and non-parenchymal liver cells and their use in investigating mechanisms of hepatotoxicity, cell signaling and ADME. Arch Toxicol.

[CR16] Grandjean P (2019). Developmental fluoride neurotoxicity: an updated review. Environ Health.

[CR17] Grandjean P, Landrigan PJ (2014). Neurobehavioural effects of developmental toxicity. Lancet Neurol.

[CR18] Green R, Lanphear B, Hornung R (2019). Association between maternal fluoride exposure during pregnancy and IQ scores in offspring in Canada. JAMA Pediatr.

[CR19] Guth S, Huser S, Roth A (2020). Toxicity of fluoride: critical evaluation of evidence for human developmental neurotoxicity in epidemiological studies, animal experiments and in vitro analyses. Arch Toxicol.

[CR20] Heindel JJ, Bates HK, Price CJ, Marr MC, Myers CB, Schwetz BA (1996). Developmental toxicity evaluation of sodium fluoride administered to rats and rabbits in drinking water. Fundam Appl Toxicol.

[CR21] Hirzy JW, Connett P, Xiang QY, Spittle BJ, Kennedy DC (2016). Developmental neurotoxicity of fluoride: a quantitative risk analysis towards establishing a safe daily dose of fluoride for children. Fluoride.

[CR22] Kupsco A, Sanchez-Guerra M, Amarasiriwardena C (2019). Prenatal manganese and cord blood mitochondrial DNA copy number: Effect modification by maternal anemic status. Environ Int.

[CR23] Leist M (2017). New animal-free concepts and test methods for developmental toxicity and peripheral neurotoxicity. Altern Lab Anim.

[CR24] Lewis RC, Meeker JD, Basu N (2018). Urinary metal concentrations among mothers and children in a Mexico City birth cohort study. Int J Hyg Environ Health.

[CR25] McPherson CA, Zhang G, Gilliam R (2018). An Evaluation of Neurotoxicity Following Fluoride Exposure from Gestational Through Adult Ages in Long-Evans Hooded Rats. Neurotox Res.

[CR26] Mullin AM, Amarasiriwardena C, Cantoral-Preciado A (2019). Maternal blood arsenic levels and associations with birth weight-for-gestational age. Environ Res.

[CR27] NHMRC (2017) National Health and Medical Research Council. Information paper – Water fluoridation: dental and other human health outcomes, report prepared by the Clinical Trials Centre at University of Sydney, NHMRC; Canberra. https://www.nhmrc.gov.au/sites/default/files/documents/reports/fluoridation-info-paper.pdf.

[CR28] NRC (2006) National Research Council; Fluoride in Drinking Water: A Scientific Review of EPA's Standards. Committee on Fluoride in Drinking Water. Board on Environmental Studies and Toxicology. Division on Earth and Life Studies. The National Academy Press, Washington DC, USA.https://doi.org/1017226/11571

[CR29] NTP (2016) National Toxicology Program. Systematic literature review on the effects of fluoride on learning and memory in animal studies. NTP Research Report 1. Research Triangle Park, NC: National Toxicology Program. 31944639

[CR30] Orta-Garcia S, Perez-Vazquez F, Gonzalez-Vega C, Varela-Silva JA, Hernandez-Gonzalez L, Perez-Maldonado I (2014). Concentrations of persistent organic pollutants (POPs) in human blood samples from Mexico City, Mexico. Sci Total Environ.

[CR31] Osmunson B, Limeback H, Neurath C (2016). Study Incapable Of Detecting IQ Loss From Fluoride. Am J Public Health.

[CR32] Pulungan ZSA, Sofro ZM, Partadiredja G (2018). Sodium fluoride does not affect the working memory and number of pyramidal cells in rat medial prefrontal cortex. Anat Sci Int.

[CR33] Rojas-Sanchez F, Kelly SA, Drake KM, Eckert GJ, Stookey GK, Dunipace AJ (1999). Fluoride intake from foods, beverages and dentifrice by young children in communities with negligibly and optimally fluoridated water: a pilot study. Community Dent Oral Epidemiol.

[CR34] Rugg-Gunn AJ, Villa AE, Buzalaf MR (2011). Contemporary biological markers of exposure to fluoride. Monogr Oral Sci.

[CR35] Soto-Barreras U, Escalante-Villalobos KY, Holguín-Loya B (2019). Effect of fluoride in drinking water on dental caries and IQ in children. Fluoride.

[CR36] Spittle B (2020). Reviews of developmental neurotoxicity by Grandjean and Guth et al. Fluoride.

[CR37] Sun H, Como PG, Downey LC, Murphy D, Ariagno RL, Rodriguez W (2015). Infant formula and neurocognitive outcomes: impact of study end-point selection. J Perinatol.

[CR38] Sutton M, Kiersey R, Farragher L, Long J (2015) Health effects of water fluoridation. An evidence review. Health Research Board, Ireland. https://www.hrb.ie/fileadmin/publications_files/Health_Effects_of_Water_Fluoridation.pdf.

[CR39] Till C, Green R, Flora D (2020). Fluoride exposure from infant formula and child IQ in a Canadian birth cohort. Environ Int.

[CR40] Valdez Jimenez L, Lopez Guzman OD, Cervantes Flores M (2017). In utero exposure to fluoride and cognitive development delay in infants. Neurotoxicology.

[CR41] Watkins DJ, Fortenberry GZ, Sanchez BN (2016). Urinary 3-phenoxybenzoic acid (3-PBA) levels among pregnant women in Mexico City: Distribution and relationships with child neurodevelopment. Environ Res.

[CR42] Watkins DJ, Sanchez BN, Tellez-Rojo MM (2017). Phthalate and bisphenol A exposure during in utero windows of susceptibility in relation to reproductive hormones and pubertal development in girls. Environ Res.

[CR43] Xiang Q, Liang Y, Chen L, et al. (2003) Effect of fluoride in drinking water on children’s intelligence. Fluoride 36(2):84–94. Erratum in Fluoride 2004;37(4):320.

